# Diabetic patients infected with helicobacter pylori have a higher Insulin Resistance Degree

**Published:** 2014

**Authors:** Jamshid Vafaeimanesh, Mohammad Bagherzadeh, Akram Heidari, Fatemeh Motii, Mahmoud Parham

**Affiliations:** 1 Clinical Research Development Center, Qom University of Medical Sciences, Qom, Iran; 2 Faculty of Medicine, Qom University of Medical Sciences, Qom, Iran

**Keywords:** Diabetes, Helicobacter pylori, Insulin Resistance

## Abstract

***Background: ***The association of H.pylori (HP) and insulin resistance degree (IR) has not been evaluated in the diabetic patients so far. In this study, we evaluated the association between HP seropositivity and the homeostatic model assessment for insulin resistance (HOMA-IR) in diabetic patients.

***Methods:*** In this study, 211 diabetic patients admitted to the endocrinology clinic of Shahid Beheshti Hospital of Qom for routine diabetic check-ups were evaluated. The patients were divided into HP^+^ and HP^-^ groups based on the seropositivity of helicobacter pylori IgG antibody. The serum H. pylori IgG antibody, blood sugar, serum insulin, HbA1c, LDL, HDL, cholesterol, triglyceride, HOMA-IR and BMI were measured.

***Results:*** The mean age of 72 HP^-^ patient was 51.5±8.3 and 139 HP+ subjects was 53.5±9 years (P=0.128). The mean HDL in HP^-^ cases was 69.2±29.2 mg/dl and for HP+ cases was 60.7±26.7 mg/dl (P=0.037). The mean serum insulin in HP^-^ was 60.97±5.64 and HP^+^ subjects was 10.12±7.72IU/ml (P=0.002). The Homa-IR degree for HP^-^ cases was 3.2±3.3 and for HP^+^ cases was 4.5±3.8 (P=0.013). There were no significant differences between these groups according to the short-term or the long-term indices of glycemic control as well as most of the diabetic risk factors or complications. The treatment type was also not significantly different between these groups.

***Conclusion: ***It seems that the HP^+^ diabetic patients require higher levels of serum insulin to reach the same degree of glycemic control compared to the HP^-^ ones.

Recognized by insulin resistance (IR), some degrees of impairment in insulin secretion and hyperglycemia, type 2 diabetes mellitus (T2DM) is a metabolic disease that is linked to different pathophysiological mechanisms. The role of inflammatory mechanisms in the pathogenesis of this disease is highlighted in recent studies (1). It is believed that inflammation may increase IR. The IR is a pathologic state in which normal insulin concentrations produce subnormal response in the peripheral tissues. In the “Sacramento Area Latino Study on Aging” (SALSA) cohort study, the seropositivity for helicobacter pylori (H. pylori) was associated with a greater rate of incident diabetes (2). The results of this study, however, are criticized by Eshraghian and Pellicano (3). As an example, they claim that the SLASA study paradoxically shows that H. pylori infection and “homeostatic model assessment for insulin resistance” (HOMA-IR) are not associated.

Nevertheless, if we assume that the H. pylori infection is a risk factor for the initiation of T2DM (4), then the arising question is “Do H. pylori infected T2DM patients have a higher degree of IR (4-6). In order to answer this question in this study, we have evaluated the relationship between the seropositivity for H. pylori and the HOMA-IR in diabetic patients who are receiving appropriate medical treatment (except insulin) for their condition.

## Methods

This study involved 211 diabetic patients who were admitted to the endocrinology clinic of Shahid Beheshti Hospital in Qom for routine diabetic check-ups. The patients with history of using H. pylori treatment, proton pump inhibitor, H2 blocker, bismuth, or insulin were excluded from the study. The smoker patients were also excluded. After 12 hours of fasting overnight, venous blood-samples were obtained and stored at 4°C. Serum was acquired by centrifugation of blood samples at 2000 r/min for 15 minutes, immediately after sampling. The serum H. pylori IgG antibody (ELISA, Padtan Elm, Iran), blood sugar, serum insulin (ELISA, DiaMetra, Italy), HbA1c, LDL, HDL, cholesterol and triglyceride were measured. Seropositivity for H. pylori was defined when the titers of higher than 30 AU/mL of IgG were detected in the serum. Multiplying the fasting glucose value (mg/dL) by serum insulin value in each person and then dividing it by 405, the HOMA-IR was calculated in this study. Also, body-mass-index (BMI) was calculated by measuring the body weight (in kg) and dividing it by the square of the height (in meters). Hypertension, coronary artery disease (CAD) and peripheral artery disease (PAD) were detected based on the medical history of the patients. The autonomous neuropathy, gastroparesis and dyspepsia were identified based on the presence or absence of symptoms such as nausea and delayed gastric emptying. The dental disease was diagnosed based on the physical examination. Using the diapason and monofilament tests, the diabetic neuropathy has been identified. The retinopathy and cataract are also diagnosed after the clinical examination of an ophthalmologist. This research was a practice compliance with the Helsinki Declaration. All the subjects were informed about the study protocol, and written consents were obtained from all participants.

**Statistical analysis: **The data were collected and analyzed by SPSS version 11. All data are reported as mean±standard deviation. The chi-square test was used to compare qualitative variables. *P-*values less than 0.05 were considered statistically significant.

## Results

Two hundred and eleven diabetic patients including 135 (64%) females and 76 (36%) males with the mean age of 52.8±8.8 years and the mean T2DM duration of 7.4±5.4 years were included in this study. The H. pylori was positive in 139 (65.9%) of patients (HP^+^ group), and negative in 72 (34.1%) of patients (HP^- ^group). The characteristics of these two groups are summarized in table 1. 

There was no statistically significant difference between these groups with respect to the IR risk factors, the complications of diabetes, and dental diseases. The lipid profile of both groups was not significantly different except serum HDL level that was slightly higher in the HP^-^ group (table 1).

The mean serum insulin and HOMA-IR were 9.0±7.2 and 4.0±3.7 µIU/mL, respectively. There was a significant difference in the level of serum insulin (HP^-^= 6.97±5.64 µIU/mL vs. HP^+^=10.12±7.72 µIU/mL, P=0.002) and HOMA-IR degree (HP^-^= 3.160±3.327 vs. HP^+^=4.484±3.781, P=0.013) between the H. pylori positive and negative groups regarding the HbA1c, FBS, or the medication types were not significantly different between these groups (table 2).

## Discussion

The T2DM is the epidemic disease of the modern ages, and IR is one of its characteristics. In this study, we evaluated the association between H. pylori infection and HOMA-IR in 211 diabetic patients who received appropriate medical treatment other than insulin. The main finding of this study was that HOMA-IR and serum insulin are significantly higher in the T2DM patients that are seropositive for H. pylori than the seronegative ones. Our results also showed that there is no significant difference in the long-term or the short-term glycemic control of patients between these groups since there was no significant difference in HbA1c, in FBS, or in the prevalence of DM complications between the HP^+^ and HP^-^ groups. Therefore, it seems that the HP^+^ patients require higher levels of serum insulin to reach the same degree of glycemic control as the HP^-^ ones.

**Table 1:** The characteristics of the type 2 diabetic patients with respect to seropositivity for Helicobacter pylori

**Parameter**	**HP** ^-^ **, n=72 (34.1%)**	**HP** ^+^ **, n=139 (65.9%)**	**P value**
**Gender** (Female/Male)	47(65.3%)/25 (34.7%)	88 (63.3%)/51 (36.7%)	0.778
**Diabetes duration** (year)	7.4±5.4	7.4±5.5	0.971
**Insulin Resistance risk factors**			
Age (year)	51.5±8.3	53.5±9.0	0.128
BMI	29.4±4.9	28.8±4.8	0.427
Waist circumference (cm)	101.46±12.31	100.69±10.82	0.971
Family history of diabetes (+/-)	53 (73.6%) /19 (26.4%)	103 (75.0%) / 36 (26.0%)	0.825
Hypertension (+/-)	32 (44.4%)/40 (55.6%)	64 (46.0%)/75 (54.0%)	0.825
Exercise degree			0.478
>150 min per week	2 (2.9%)	11 (7.9%)	
30-150 min per week	4 (5.7%)	10 (7.2%)	
<30 min per week	15 (21.4%)	26 (18.7%)	
No	51 (72.9%)	92 (66.2%)	
**Diabetes Complications **			
Neuropathy			0.165
Motor	2 (2.8%)	3 (2.2%)	
Sensory	31 (43.1%)	79 (56.8%)	
No	39 (54.1%)	57 (41%)	
Autonomous neuropathy (+/-)	44 (61.1%)/28 (38.9%)	74 (53.2%)/65 (46.8%)	0.275
Gastroparesia (+/-)	16 (22.2%)/56 (77.8%)	34 (24.5%)/105 (75.5%)	0.717
Nephropathy			0.795
Macroalbuminuria	5 (6.9%)	13 (9.4%)	
Microalbuminuria	19 (26.4%)	33 (23.7%)	
No	48 (66.7%)	93 (66.9%)	
CAD (+/-)	16 (22.2%)/56 (77.8%)	38 (27.3%)/101 (72.7%)	0.419
PAD (+/-)	17 (23.6%)/55 (76.4%)	22 (15.8%)/117 (84.2%)	0.167
CVA (+/-)	6 (8.3%)/66 (91.7%)	14 (10.1%)/125 (89.9%)	0.683
Retinopathy			0.664
Proliferative	5 (6.9%)	6 (4.3%)	
Non-proliferative	22 (30.6%)	40 (28.8%)	
No	45 (62.5%)	93 (66.9%)	
Cataract (+/-)	31 (43.1%)/41 (56.9%)	42 (30.7%)/95 (69.3%)	0.074
Dental diseases (+/-)	6 (8.3%)/66 (91.7%)	16 (11.7%)/121 (88.3%)	0.454
**Lipid profile**			
HDL (mg/dL)	69.2±29.2	60.7±26.7	0.037
LDL (mg/dL)	107.1±43.2	116.0±51.7	0.212
TG (mg/dL)	224.2±100.2	229.3±114.6	0.747
Cholesterol (mg/dL)	205.1±63.2	207.3±67.4	0.820

**Table 2:** Insulin resistance, glycemic control and medication type in type 2 diabetic patients with respect to seropositivity for Helicobacter pylori

**Parameter**	**HP** ^-^	**HP** ^+^
**Serum insulin** (µIU/ml)	6.97±5.64	10.12±7.72
**HOMA-IR degree**	3.2±3.3	4.45±3.8
**FBS** (mg/ dL)	173.43±61.32	180.12±64.27
**HbA1c** (%)	8.08±1.32	8.11±1.67
**Type of Treatment**		
Non-pharmacologic	2 (2.8%)	6 (4.3%)
One type of Medication		
Metformin	7 (9.7%)	10 (7.2%)
Glyburide (Glibenclamide)	6 (8.3%)	22 (15.8%)
Acarbose	0 (0%)	1 (0.7%)
Two types of Medication		
Metformin + Glyburide	22 (30.6%)	52 (37.4%)
Metformin + Pioglitazone	1 (1.4%)	3 (2.2%)
Metformin + Acarbose	2 (2.8%)	7 (5.0%)
Glyburide + Pioglitazone	3 (4.2%)	1 (0.7%)
Glyburide + Acarbose	0 (0%)	4 (2.9%)
Pioglitazone + Acarbose	1 (1.4%)	0 (0%)
Three types of Medication		
Metformin + Glyburide + Pioglitazone	7 (9.7%)	4 (2.9%)
Metformin + Glyburide + Acarbose	9 (12.5%)	15 (10.8%)
Metformin + Pioglitazone + Acarbose	1 (1.4%)	2 (1.4%)
Four types of Medication		
Metformin+Glyburide+Pioglitazone+Acarbose	11 (15.3%)	11 (7.9%)
Repaglinide+Glyburide+Pioglitazone+Acarbose	0 (0%)	1 (0.7%)

p=0.002

p=0.013

The association between the IR and H. pylori infection among the otherwise healthy individuals has been issued in several previous studies, but as far as we know, this relation has not been evaluated in diabetic patients so far. In addition, there have been a limited number of these studies and no consensus among them (2, 4, 7-19). A recent meta-analysis, for instance, shows that H. pylori infection is more frequent in diabetic patients (20). Another meta-analysis, however, found that it is impractical to analyze the association between H. Pylori infection an IR, because of the biasing effect of the small percentage of patients which could be included in the study (21). Consequently, further studies are required in this regard (22). Overall, although the positive association of these two conditions is not a fact yet, the general trend is towards it (21, 22). Most of those studies that showed no association between H. pylori infection and IR were not specifically designed for this purpose. For instance, serum insulin but not HOMA-IR is considered as the index of IR in the Gillum et al.'s study. Naja et al. were criticized for not considering the previous history of H. pylori treatment or the use of insulin, anti-acid and bismuth medications as the exclusion criteria (23). These methodological flaws do not apply to our study and our results correlate with the above mentioned trend.

Jeon et al. proposed the possible role of altered gut microbiota in the pathogenesis of insulin resistance and T2DM (2). Concentrations of circulating lipopolysaccharides (a part of the bacterial cell wall) have been reported to be higher in obese patients with T2DM than in non-diabetic thin individuals and correlate with insulin resistance severity (24). Serum lipopolyaccharides originate from the gastrointestinal tract and their levels increase after eating a meal rich of lipids. H. pylori gastritis and its role in mucosal activation of innate immunity and upregulation of IL-1β is also suggested in the pathogenesis of IR (25). H.pylori gastritis and its effects on ghrelin may also affect appetite and insulin sensitivity (26). 

It seems that H. pylori eradication treatment may be helpful in lowering the IR in T2DM patients. Nevertheless, there is no agreement among researchers in this regard. Gen et al. showed that the H. pylori eradication reduced the HOMA-IR in dyspeptic non-diabetic patients (13). After one year of follow up, Park et al., however, showed that HOMA-IR was not significantly different in patients receiving appropriate medication for the eradication of H. Pylori compared to the control group (27). In our study, medication type and short-term or long-term glycemic control were not different between the HP^+^ and HP^-^ groups, which is in agreement the with findings of other studies (27). Yet, we cannot exclude the possibility that the drug dose may be higher in the HP^+^ group. 

According to previous studies, HP^+^ non-diabetic individuals compared with the HP^-^ ones have a higher HbA1c level (28). The eradication of H. pylori, however, does not change the HbA1c level in T2DM patients, which was predictable since these patients receive appropriate medications (29). Our findings are also in agreement with these results.

In our study, most of the known risk factors of the T2DM are the same between HP^+^ and HP^-^ groups. It seems that the systematic bias is minimized in this study. On the other hand, we are aware of the limitations of epidemiological indices that we have used for the detection of H. pylori infection or the calculation of IR degree. Consequently, although our study shows the association between H. pylori infection and HOMA-IR in diabetic patients, we suggest better indices to be used for the detection of H. pylori infection or the calculation of IR in the future studies in this regard. We also suggest that the association between the dose of T2DM medications and the IR needs to be evaluated in the future studies.

## Notes

**Funding: **This paper is a general physician’s thesis financially supported by Qom University of Medical Sciences, Qom, Iran. (Grant number: 90252).
